# Reconstruction of Hair-bearing Areas of the Head and Face in Patients With Burns

**Published:** 2008-08-11

**Authors:** Shahram Nazerani, Mohammad Hosein Kalantar Motamedi

**Affiliations:** ^a^ Department of Surgery, Iran University of Medical Sciences, Tehran, IR Iran; ^b^ Trauma Research Center, Baqiyatallah Medical Sciences University, and Azad University of Medical Sciences, Tehran, IR Iran

## Abstract

**Aim:** Tissue expansion is a well-documented surgical option. In patients with burns with defects in hair-bearing areas, the paucity of hair-bearing skin and donor-site problems presents a complicated issue. Herein we share our results in 10 patients with scalp and facial burns, requesting the reconstruction of hair-bearing areas and address important issues to bear in mind when undertaking this procedure. **Materials and Methods:** Ten patients with scalp, face, and neck burns were treated with tissue expanders for generating hair in hair-bearing areas, and were later treated with free or pedicle flaps. Expanders were placed in the temporal and/or the occipital or supraclavicular areas for 80 to 130 days depending on defect size. Tissue expansion was done slowly at weekly intervals; after completing the predicted expansion, a wait period of 3 to 4 weeks for the expanded tissue to rest was observed before the second operation. To reconstruct the scalp and facial hair-bearing areas, 2 free scalp flaps and 8 rotation flaps were prepared and transferred to the head, neck, and face in these patients. **Results:** Ten burn patients aged 20 to 35 years (mean = 28 years) (9 men and 1 woman) were treated with expanders followed by flaps. Defects ranged in size from 5 to 20 cm^2^. Complications, mainly infection of the tissue-expander pocket, seroma formation, and partial flap loss (1 patient) were encountered. **Conclusion:** Tissue expansion is a useful method for reconstruction of hair-bearing-area defects of the scalp, neck, or face, with good cosmetic results. Expansion is slightly more difficult in patients with burn scars and requires greater attention to technical details to prevent untoward complications. However, hair-bearing area reconstruction in burn and trauma patients done with expanded scalp skin has several major advantages: (1) The ability to close the donor site primarily, (2) expanded scalp skin has less hair follicles per square centimeter, (3) thinner skin provides a near perfect match to the facial skin, and (4) expanded skin can be transferred as a free or pedicle flap and can be even used to reconstruct multiple areas, such as eyebrow and cheek and mustache, simultaneously.

In Middle Eastern cultures hair-bearing areas of face are regarded as signs of manhood, and there are many patients who request reconstruction of these areas. Because the hair-bearing areas of the scalp have limited donor capacity, tissue expansion generates excess skin by stretching the skin elements and the end result is expanded skin that is much thinner than the normal skin. The skin elements are stretched and thinned. This result of tissue expansion is an added benefit in hair-bearing areas because the expanded skin has less hair follicles per square centimeter; therefore in terms of hair density, it is better matched to the facial skin, which has fewer hair follicles per square centimeter than the scalp. Another advantage is the ability to close the scalp donor site without tension. The tissue expansion for scalp is rather straightforward and some sample cases are presented. But reconstruction of the mustache, beard, and eyebrow are rather more time-consuming and need several operations and attention to details. However, the end result of expanded tissue with less hair follicles per square centimeter and the primary closure of the donor area without alopecia are benefits of this method.

## MATERIALS AND METHODS

**Figure 1:**
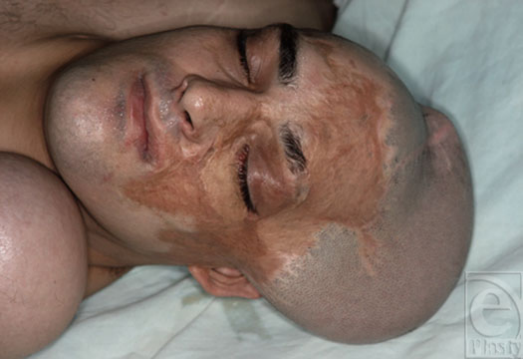
Patient with scars in eyebrow, cheek, and mustache areas, who requested reconstruction of theses areas.

**Figure 2:**
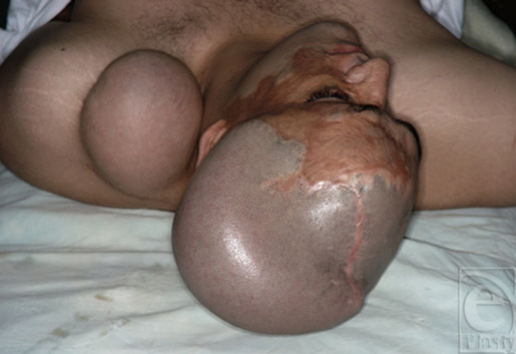
The patient with 2 tissue expanders in the supraclavicular and head areas to reconstruct the forehead and scalp.

**Figure 3:**
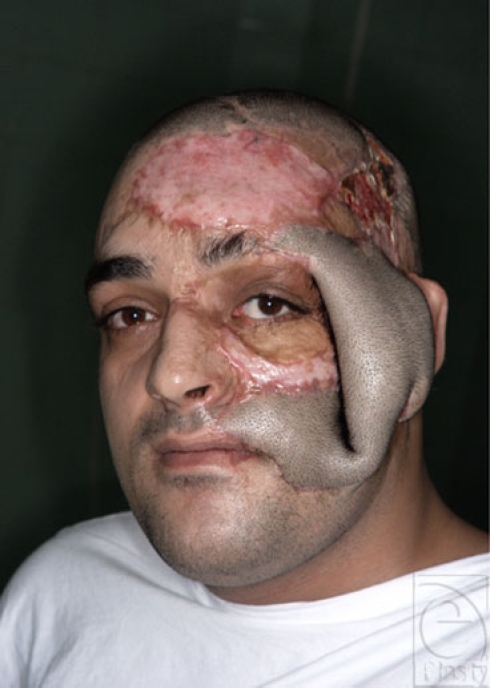
The donor site of the rotated flap, which was loosely approximated and left to granulate because the remaining flap had been scheduled to be returned.

**Figure 4:**
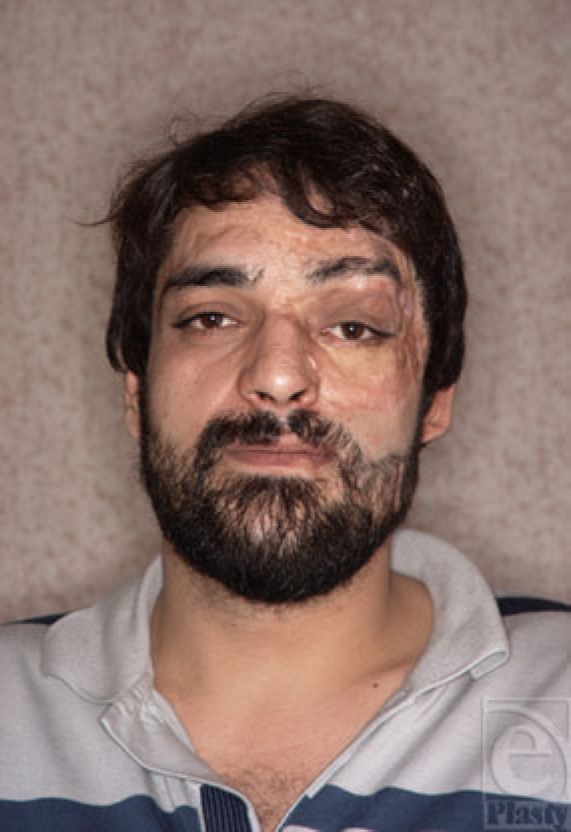
The end result of forehead, mustache, cheek, and eyebrow reconstruction.

**Figure 5:**
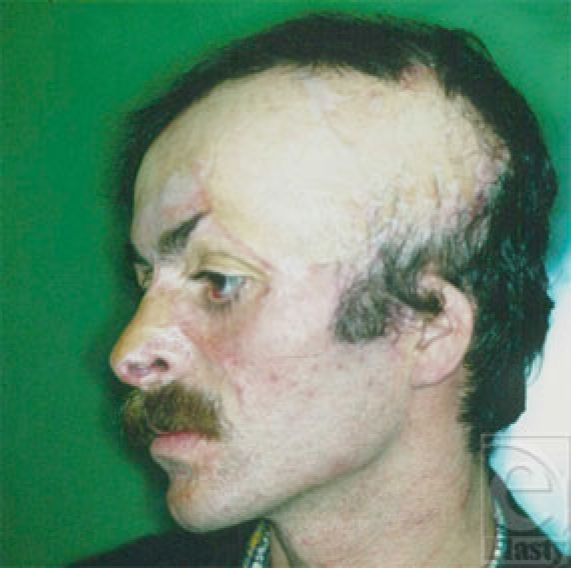
A large scalp defect of a war victim.

**Figure 6:**
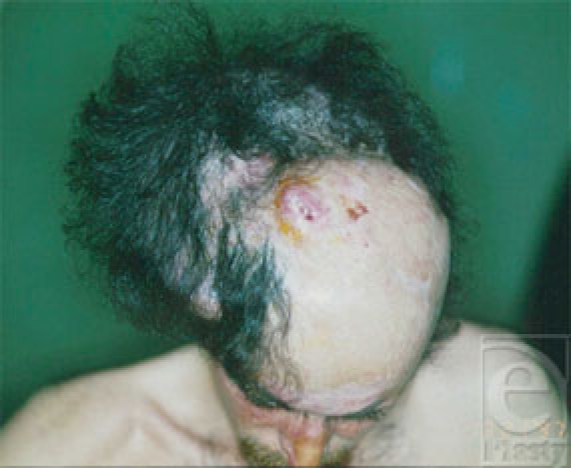
An 800-ml banana-shaped tissue expander was placed under the scalp and expanded to its maximum.

**Figure 7:**
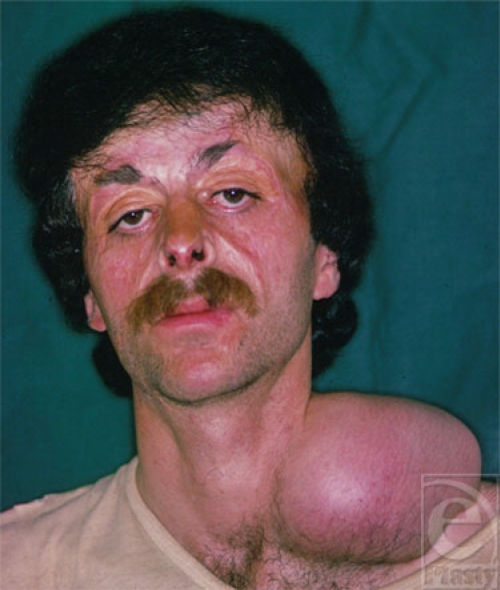
The end result of scalp and the supraclavicular expanded tissue ready for forehead reconstruction.

**Figure 8:**
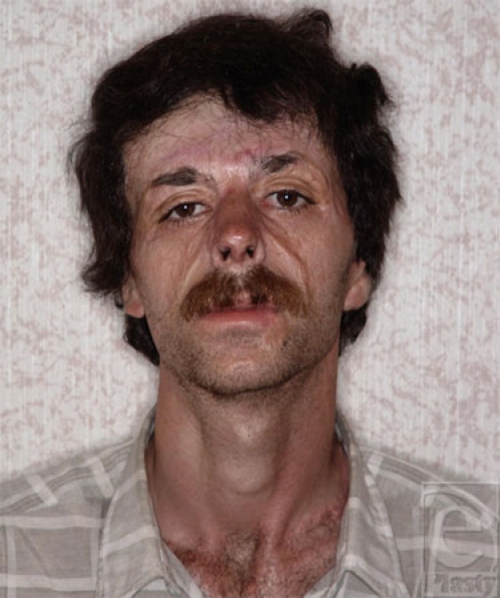
The end result of scalp and forehead reconstruction.

Ten patients with scalp, face, and neck burns were treated with tissue expanders to generate hair-bearing areas, followed by free or pedicle flaps (Figs 1–10). One or 2 expanders were placed under the scalp in the temporal and/or the occipital areas or in the supraclavicular area for 80 to 130 days depending on defect size to simultaneously reconstruct hair-bearing areas of the head or face. The most important point in inserting an expander is that first the insertion incision should be as far as possible from the scar. Second, when the incision must be made near the scar it should be, if possible, perpendicular to the longest axis of the expansion site to prevent dehiscence of the insertion wound and extrusion of the expander. All patients underwent reconstruction of the soft-tissue defects with the expanded scalp soft tissue in a 2-stage operation. In the first stage, a tissue expander (cylindrical or banana shaped, size 150 to 800 ml) was implanted into the skin to achieve a skin soft-tissue expansion. Our protocol in tissue expansion is a slow expansion at weekly intervals and after completing the predicted expansion a wait period of 3 to 4 weeks for the expanded tissue to rest is observed before the second-stage operation. After a sufficient skin expansion (8 cm × 5 cm to 25 cm × 23 cm) was made by weekly saline injection, a properly designed skin flap was taken and transferred to reconstruct the scalp soft-tissue defect in the second stage of the operation. All the scalp and hair-bearing area defects, which ranged in size from 1 cm × 5 cm to 20 cm × 10 cm, were treated. To reconstruct the scalp and facial hair-bearing areas, 2 free scalp flaps and 8 rotation flaps were prepared and transferred to the head, neck, and face.

## RESULTS

The patients aged 20 to 35 years (mean = 28 years) were treated with tissue expanders followed by flaps. There were 9 men and 1 woman. Defects ranged in size from 5 to 20 cm^2^ (Table 1). The hair grew well in all flaps and the scars were hidden with a satisfactory appearance. There were several complications, including mainly infection of the tissue expander pocket, seroma formation, and partial flap loss. The most common complication was seroma formation under the donor site. We did not encounter any exposed tissue expanders. Reconstruction of the scalp and facial soft-tissue defects with expanders is an ideal method, provided technical details are observed.

## DISCUSSION

Deep burns of the scalp responsible for alopecia posed a great surgical challenge until the use of tissue expansion in the 1980s. Tissue expanders have been used in our unit for more than 15 years, especially for head and neck reconstruction after burns. Out of 60 patients admitted to our clinic with various facial and scalp burns requiring tissue expansion for reconstruction, 10 patients required hair-bearing area reconstruction. The incidence of postsurgical complications is decreasing with improvements in surgical technique; types of expanders (size, location, etc); and design of flaps.

Reconstruction of soft-tissue defects in the head and neck is best accomplished using similar tissue.^1^ Large scalp defects in the setting of previous excisions, irradiation, or burns are difficult to reconstruct and rehabilitate. In all of these cases, the ability to transfer expanded tissue has improved the functional and cosmetic outcomes.^2^

The overall complication rate was 10%, which is the same as the literature reported; however, the noteworthy point is we have had no expander extrusion or exposure, but the reported literature rate is between 3% and 5%.

The proper way to employ this technique for scalp reconstruction usually presents a challenge to the surgeon, especially in the case of a “sideburn” scenario or a large lesion, as with, for example, hemiscalp alopecia.^3^,^4^ Application of a dermal regeneration template with a thin split-thickness skin graft followed by sequential hair micrografting is a viable alternative for hair-bearing scalp reconstruction in cases where traditional methods (eg, tissue expansion, microvascular free tissue transfer) are unavailable or undesirable.^5^ Severe postburn scarring of the lower face in the adult male may cause loss of mustache and beard, which in eastern cultures is undesirable. The skin incision for insertion of a tissue expander under a pocket of skin, be it scalp or neck skin, should be perpendicular to the longest axis of expansion, which has the strongest pull on surrounding tissues. The reason being that with stretching of the skin a perpendicular incision will be tightly closed, whereas a parallel incision will open up.

The skin incision for inserting tissue expander was usually about 5 to 10 cm away from the actual pocket, but the important point is the perpendicularity of incision to the longest axis of the pocket.

The bipedicle scalp flap or “visor flap” is used periodically for reconstruction. Our results indicate that the scalp flap is the ideal match for reconstruction of the male hair-bearing areas of the face only after expansion and the thinning of the scalp skin. The scar aspect is very satisfactory.^6^ The major advantage of this procedure is that the donor site morbidity and alopecia associated with the classic visor flap are virtually eliminated.^7^–^9^ Our results show that tissue expansion is a simple, safe, and efficient technique for hair-bearing area reconstruction. The expanded tissue can be transferred as a free or pedicle flap and the first advantage is the donor-site closure, the second being the thinning of the scalp flap obtained by expansion, which helps make facial skin match natural skin.

## Figures and Tables

**Figure 9a:**
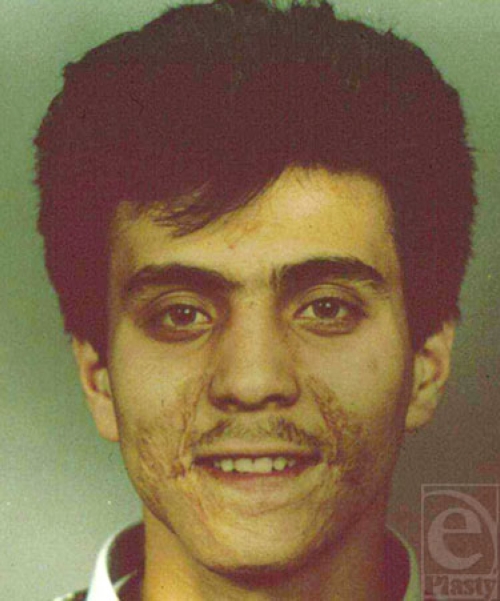
A beard area defect.

**Figure 9b:**
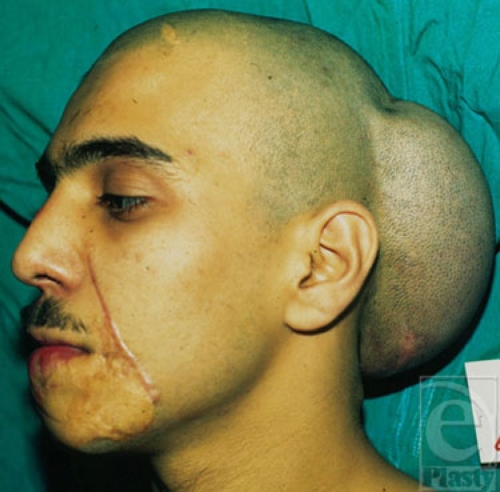
Expanded scalp ready for transfer, which was transferred as a free flap with temporal pedicle as its nutrient vessels.

**Figure 9c:**
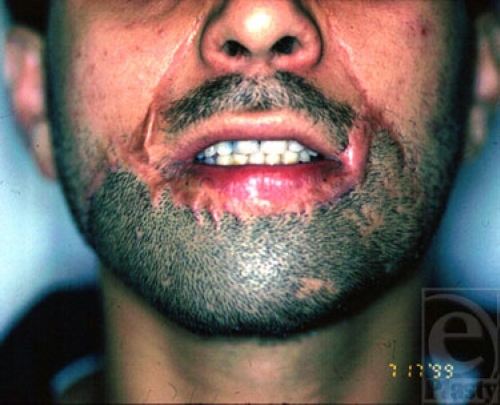
Appearance of free vascularized expanded scalp transfer, 6 months after operation.

**Figure 10a:**
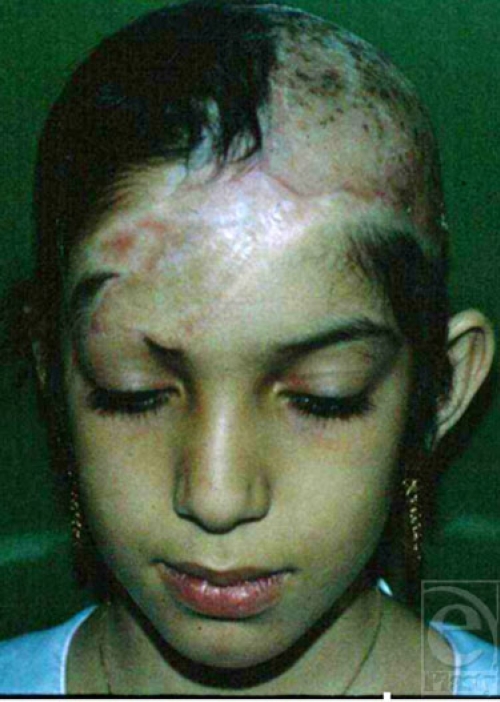
An avulsed scalp, skin-grafted following an accident in an amusement park.

**Figure 10 b and c:**
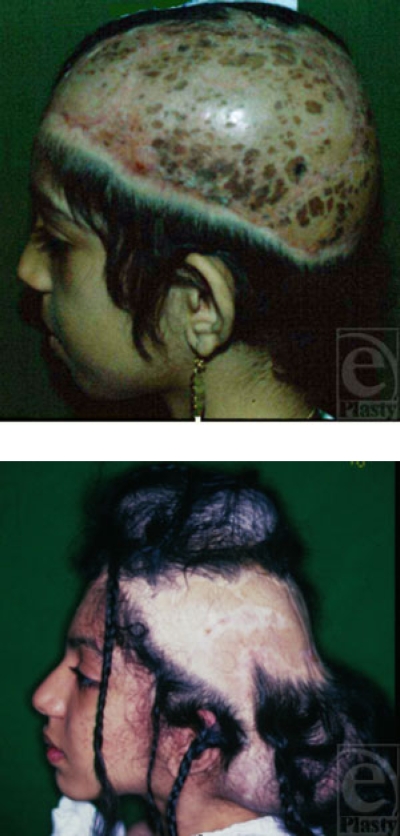
Expanded scalp skin via 2 tissue expanders placed at the right temporoparietal and occipital areas was used to treat this patient.

**Figure 10d:**
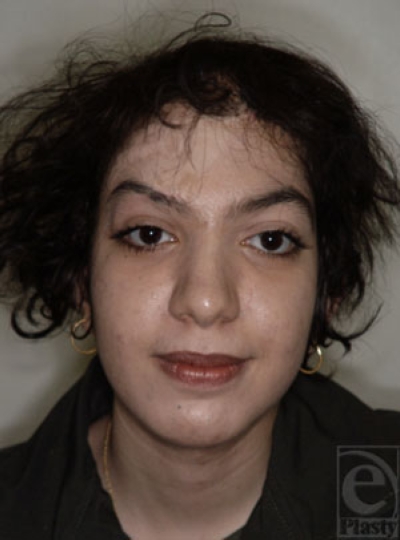
The end result of scalp reconstruction.

**Table 1:** Data concerning diagnosis and treatment of 10 patients with expanders followed by flaps to reconstruct hair-bearing areas of the head and face after burn injuries

**Patient no.**	**Diagnosis**	**First operation**	**Second operation**	**Third operation**	**Fourth operation**	**Complications**	**Operations**
1	Scars on face, nose, and eyebrow	Tissue expander—scalp	Expander was removed; scalp flap was used for eyebrow reconstruction	Flap was severed, scar removed, chin-defatted flap of the nose with fat graft, dorsal side Z-plasty, brow lift	Scar revision		4
2	Brow defect	Tissue expander—scalp	Expander removal, free scalp flap for brow reconstruction—Z-plasty	Brow flap, eyelid defatted, lateral canthus deepened VY plasty	Frontal flap for coverage of the upper eyelid		4
3	Facial scar	Tissue expander placed under parietal scalp area	Expander removal, scalp repair—free scalp flap to cover chin and cheek. Face lift and scar resection	Chin scar was removed, nasolabial area facial scars were removed, commisuroplasty and multiple Z plasty were performed		Postoperative bleeding	3
4	Scalp necrosis	Debridement of necrotic tissue and skin	Tissue expander—scalp	Tissue expander removed and scalp was moved anteriorly		Seroma under flap	3
5	Brow defect	Tissue expander—scalp	Expander removed, scalp flap for eyebrow on temporal vessels	Flap was freed, necrotic skin removed	Microhair transplant was for eyebrow under forehead. Scar revised	Failed; artery tight tunnel	4
6	Scar on temporoparietal area	Tissue expander insertion under occipital and forehead area	Removal of tissue expanders. Forehead and scalp scars were removed and scalp covered the defect	subSMAS facelift— facial muscles elevated, excess scar removed. Z-plasty, 3 slips of goretex to elevate facial muscles, lower eyelid debulked and fixed transversely			3
7	Alopecia of scalp, bilateral	Tissue expander inserted under the occipital area	Expander removal, scalp repaired. Another rectangular tissue expander placed in the suprascapular area	Expander removal, multiple Z-plasty and derambrasion of chin and cheek of forehead. Z-plasty of earlobe and tragus and scar excision	Scalp reconstruction, alopecia covered, forehead and nose scars removed	Infection of tissue expander	4
8	Facial and forehead scars	Two tissue expanders placed on temporal scalp and supraclavicular areas	Tissue expanders removed, forehead and lower eyelid scars removed, scalp flap turned down, and eyebrow, beard, and mustache reconstruction	Scar revision, face and nose Z-plasty, dermabrasion, defatting of the flap. Z-plasty of ear and defatting of eyebrow			3
9	Head, neck, and face scars	Tissue expander inserted—scalp	Tissue expander was removed and flap was transferred to the neck. Scar removal, flap transferred, defatted and Z-plasty performed	Ears' scars were released	Liposcultpture of the neck and flap defatting		3
10	Facial scar	Tissue expander— scalp	Expander removed and visor flap transferred	Flap severed and facial reconstruction	Scar revision		4
